# Supplementary material for: Microbial associates of the elm leaf beetle: uncovering the absence of resident bacteria and the influence of fungi on insect performance
Source: Appl Environ Microbiol. 2024 Jan 5;90(1):e01057-23. doi: 10.1128/aem.01057-23 (PMC10807431; doi:10.1128/aem.01057-23)
Supplement: Supplemental figures — Fig. S1 to S3. [file aem.01057-23-s0001.pdf]

SUPPLEMENTARY FIGURES

Microbial Associates of the Elm Leaf Beetle: Uncovering the Absence of Resident Bacteria and the Influence of Fungi on Insect Performance

Johanna Schott[a], Juliette Rakei[a], Mitja Remus-Emsermann[b], Paul Johnston[c,d], Susan Mbedi[c,e], Sarah Sparmann[c,d], Monika Hilker[a], Luis R. Paniagua Voirol[b]#

Overview

FIGURE S1. Overview of bacteria via PCR targeting the 16S rRNA gene

FIGURE S2. Library size comparison across samples

FIGURE S3. Abundance of the 20 most prevalent bacterial genera from negative controls across samples

FIGURE S1.

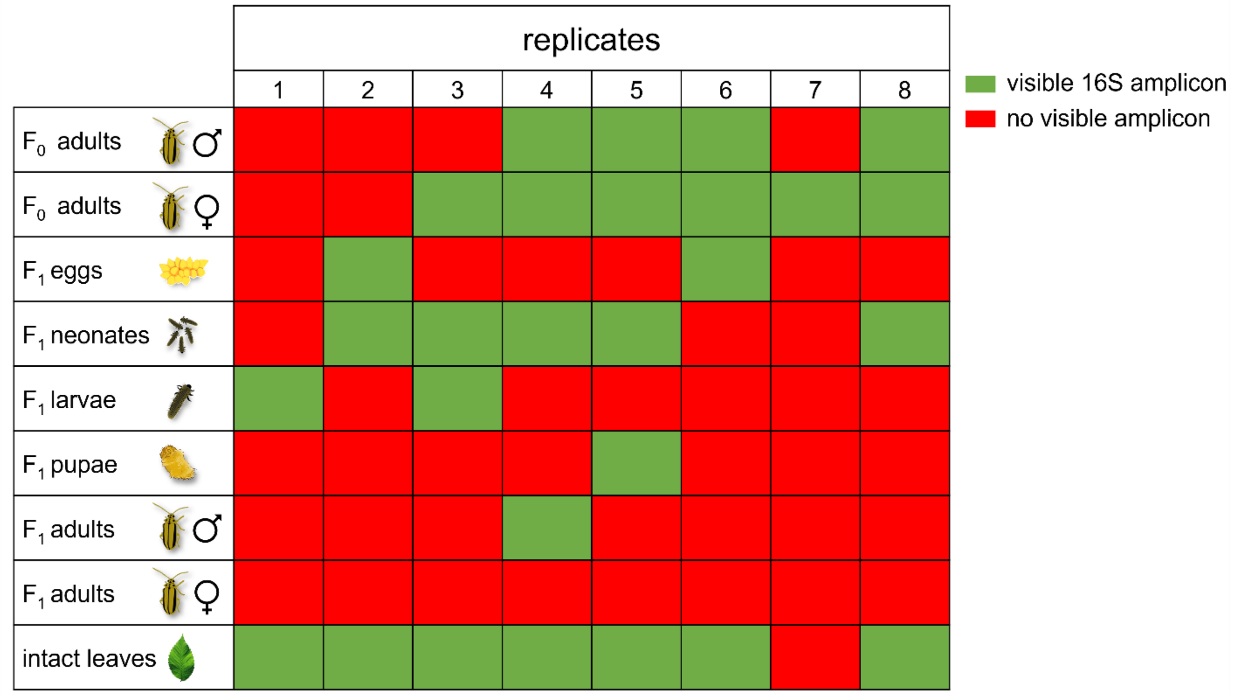

Figure S1. Overview of bacteria detection in samples of the elm leaf beetle (*Xanthogaleruca luteola*) and elm (*Ulmus minor*) leaves via PCR targeting the 16S rRNA gene. Bacterial 16S rRNA gene amplification was performed on samples collected from F_0 adults, their offspring across all life stages, and intact elm leaves. Universal primers 515F (5'-GTGYCAGCMGCCGCGGTAA-3') and 806R (5'-GGACTACNVGGGTWTCTAAT-3') recommended by the Earth Microbiome Project (EMP: https://earthmicrobiome.org) were used for the PCR amplification. Each reaction consisted of a 50 μL volume containing 50 ng of DNA template. The PCR cycles were performed according to the protocol described for the culture-dependent analysis. Amplified products (amplicons) were visualized on a 1% agarose gel stained with ethidium bromide. Samples that showed a signal of successful amplification (visible bands) are depicted in green, while those that provided no signal of amplification (no visible bands) are shown in red.

FIGURE S2.

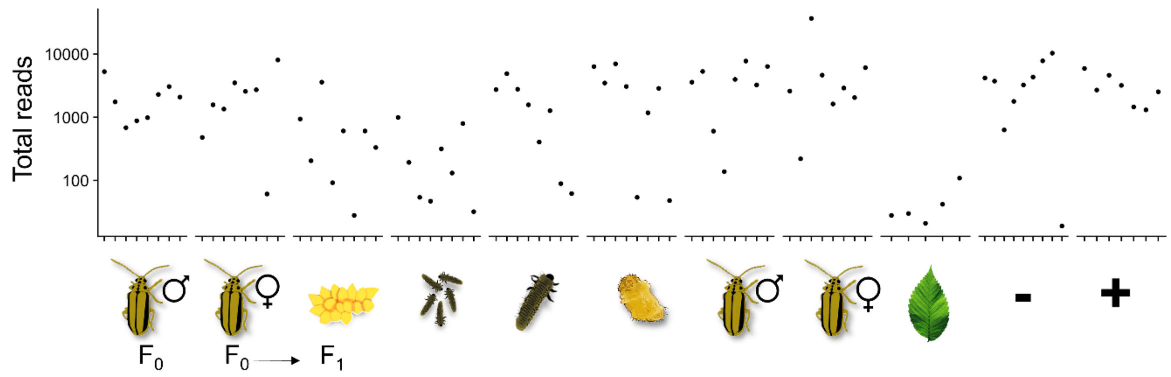

Figure S2. Library size comparison across samples. MiSeq sequencing of the bacterial 16S rRNA gene was conducted on samples from elm leaf beetle (*Xanthogaleruca luteola*) F0 adults, their offspring across all life stages, intact elm (*Ulmus minor*) leaves, and negative and positive controls. Each bar depicts an individual sample. Of the initially analysed eight leaf samples, three were excluded from further analysis due to yielding fewer than 10 reads after chloroplast read exclusion. Refer to the Materials and Methods section for detailed sequencing procedures.

FIGURE S3.

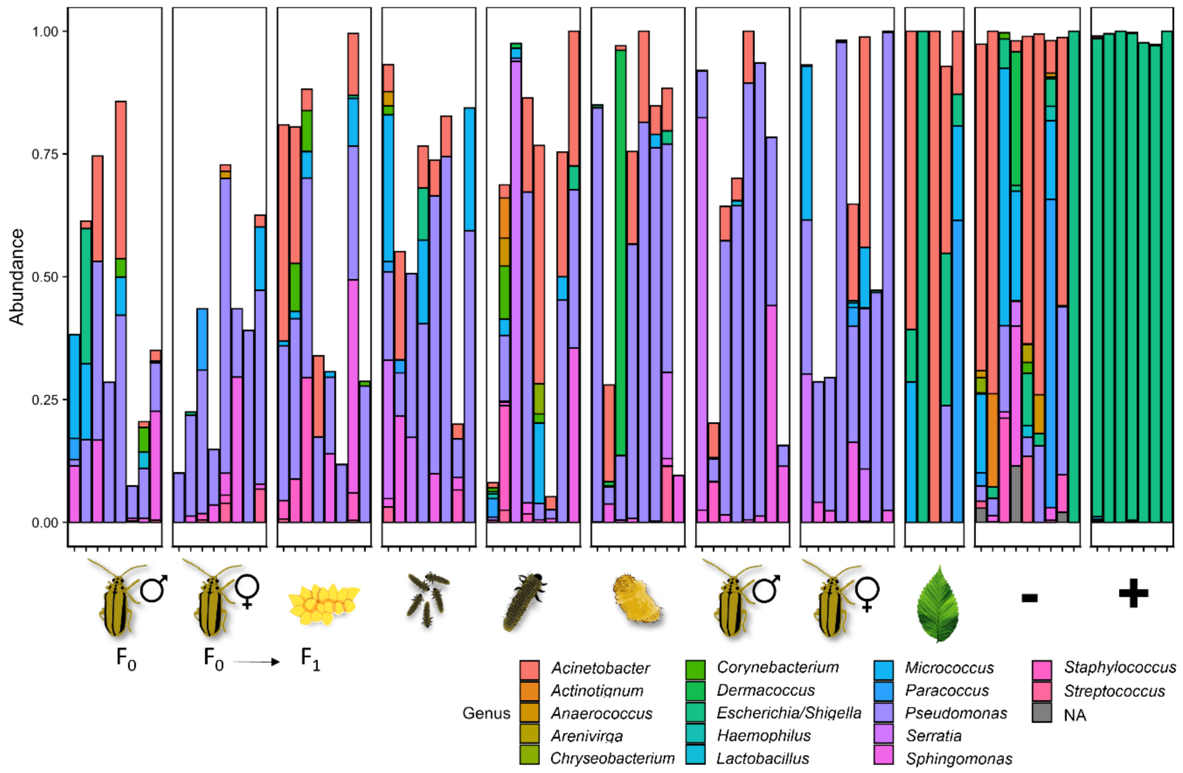

Figure S3. Abundance of the 20 most prevalent bacterial genera from negative controls across samples. MiSeq sequencing of the bacterial 16S rRNA gene was conducted on samples from elm leaf beetle (*Xanthogaleruca luteola*) F0 adults, their offspring across all life stages, intact elm (*Ulmus minor*) leaves, and negative and positive controls. The figure depicts the relative abundance across experimental samples of the 20 most prevalent bacterial taxa identified in the negative controls. Note that two taxa lacked genus classification (NA). Each bar represents an individual sample. Three of the original eight leaf samples were excluded from the analysis due to yielding fewer than 10 reads. Refer to the Materials and Methods section for detailed sequencing procedures.
